# Modern contraceptive utilization and associated factors among married and cohabiting women in Papua New Guinea: a population-based cross-sectional study

**DOI:** 10.1186/s40834-020-00125-6

**Published:** 2020-11-18

**Authors:** Abdul-Aziz Seidu, Ebenezer Agbaglo, Louis Kobina Dadzie, Bright Opoku Ahinkorah, Edward Kwabena Ameyaw, Justice Kanor Tetteh, Sanni Yaya

**Affiliations:** 1 Department of Population and Health, University of Cape Coast, Cape Coast, Ghana; 2 College of Public Health, Medical and Veterinary Sciences, James Cook University, Townsville, Queensland Australia; 3 Department of English, University of Cape Coast, Cape Coast, Ghana; 4 School of Public Health, Faculty of Health, University of Technology Sydney, Sydney, Australia; 5 School of International Development and Global Studies, University of Ottawa, Ottawa, Canada; 6 The George Institute for Global Health, The University of Oxford, Oxford, UK

**Keywords:** Contraceptives, Papua New Guinea, Public health, Global Health, Women

## Abstract

**Background:**

Universal access to family planning has been emphasized by the international development agenda, as evident in the Sustainable Development Goal 3.7. This notwithstanding, the use of modern contraceptives has been minimal in low- and middle-income countries, especially in Papua New Guinea. In view of this, we investigated the factors associated with the use of modern contraceptives and the associated factors among married and cohabiting women in Papua New Guinea.

**Methods:**

The study utilised the Demographic and Health Survey data of 2345 women in sexual unions in Papua New Guinea. We employed a descriptive and binary logistic regression analyses. We presented the results as crude Odds Ratios (COR) and adjusted Odds Ratios (AOR), with 95% confidence intervals (CI) signifying level of precision. Level of statistical significance was set at *p* < 0.05.

**Results:**

We found that 74.4% of the women were using modern contraceptives ranging from injectables (44.5%) to other modern methods (0.23%). Women aged 15–19 [AOR = 7.425, 95% CI = 2.853, 19.32], residents of the Highland region [AOR = 1.521, 95% CI =1.086, 2.131], self-employed women in the agricultural sector [AOR = 1.710, 95% CI = 1.218, 2.400], and women who listened to radio at least once a week [AOR = 1.409, 95% CI = 1.048, 1.895] had higher odds of modern contraceptive usage. However, women in the Islands region [AOR = 0.291, 95% CI = 0.224, 0.377], women whose husbands had higher education [AOR = 0.531,95%CI = 0.318,0.886], women in professional/technical/managerial work [AOR = 0.643, 95% CI = 0.420, 0.986], and those with no child [AOR = 0.213, CI = 0.0498,0.911] had lower odds of modern contraceptive use.

**Conclusion:**

Out of the 2345 participants, we found that majority of them were using modern contraceptives and the commonly used modern contraceptive was injectables. Age, region of residence, partner's education, employment, partner's desire for children, and frequency of listening to radio are associated with modern contraceptive usage. Tailored reproductive healthcare should be developed for women who are disadvantaged when it comes to the usage of modern contraceptives in order to boost modern contraceptive use among them. Further investigation is needed to unravel the motivation for the high usage of injectables among married and cohabiting women in Papua New Guinea.

## Background

Making access to family planning universal has been an important component of the global development agenda. The International Conference on Population and Development Program of Action, for instance, declared it as a fundamental human right [1–3]. More importantly, the target 3.7 of the Sustainable Development Goals (SDGs) emphasises “the universal access to sexual and reproductive health-care services, including family planning” [4]. In contemporary society, modern contraception is the most preferred option and includes sterilization, intrauterine devices and systems, subdermal implants, oral contraceptives, condoms, injectables, emergency contraceptive pills, patches, diaphragm and cervical caps, spermicidal agents, vaginal rings, and sponge [5]. Utilisation of modern contraception allows couples to determine the number and spacing of their pregnancies. It additionally ensures good health for both mothers and children, by decreasing mortality and morbidity induced by unwanted pregnancies [6–9]. Further, increasing contraceptive use reduces fertility, which indirectly leads to poverty reduction [10].

There has been a flurry of research on modern contraception use all over the world, especially in low- and middle-income countries. Part of such research has focused mainly on reasons for non-use of contraception [11] and factors that positively influence the use of contraception, with others have focused specifically on the influence of social support and parity [12], internal migration, and knowledge of contraceptive use on the use of contraception [13]. In terms of geography, such research has focused on countries such as India [14], twenty-seven sub-Saharan African countries [15], Gambia [16], and Ethiopia [17]. Research by Adebowale et al. [11], for instance, revealed being married more than once and husband’s non-approval as barriers to contraception use in Burkina Faso. Women’s education [15], parity [17], age, religion, and type of marriage [18] have also been identified as predictors of modern contraceptive use among women.

In the Pacific, previous related studies basically focused on follow-up of a contraceptive outreach program [19], access to contraception and family planning [20], adolescent fertility and family planning [21], and costs and benefits of reducing unmet need for contraception [10]. To be more specific, in Papua New Guinea, previous related studies focused on women’s perspective of family planning [22], and impact of the contraceptive implant on maternal and neonatal mortality [23], but failed to reveal the factors associated with modern contraception use. In the present study, we investigated the factors associated with the use of modern contraception in Papua New Guinea. This study is desirable, given that, as previous studies reveal, Papua New Guinea records low use of modern contraceptive, which has led to a high maternal mortality ratio, estimated to be around 57.3% per 100,000 live births [24]. Thus, revealing factors associated with modern contraception use in Papua New Guinea, as this study aims to do, will not only extend the geographical coverage of research of this kind, but also help health intervention programs aimed at reducing maternal mortality due to unintended pregnancies and abortions in the country.

## Materials and methods

### Data source

The data used for this study forms part of the 2016–2018 Papua New Guinea Demographic and Health Survey (PDHS), which was collected from October 2016 to December 2018. The survey adopted a two-staged stratified sampling technique. Before the sampling, the provinces in the country were apportioned into urban and rural areas, which yielded 43 strata; however, the National Capital District had only urban areas. A two-staged sampling procedure was used to sample census units (CUs) from each stratum. Stage 1 involved the selection of 800 CUs. This was done through probability proportional to CU size. The second stage saw the systematic selection of 24 households from each cluster through probability sampling, and this yielded a total of 19,200 households. For this study, we focused on women in sexual unions, with “sexual union” defined as marriage or cohabitation, and such women numbered 2345, all of which had complete information on the variables the present study is interested in. Details of the methodology, pretesting, training of field workers, the sampling design, and selection are available in the PDHS final report [25] which is also available online at: https://dhsprogram.com/publications/publication-fr364-dhs-final-reports.cfm.

### Variables

#### Outcome variable

The outcome variable was modern contraceptive utilization. A woman was deemed to be a modern contraceptive user if she uses at least one of the following methods: female sterilization, male sterilization, IUD, injectable, implants, pills, male condom, female condom, emergency contraception, and standard days method. On the other hand, non-users are those who resort to the use of traditional methods such as rhythm method, lactation amenorrhea method, and withdrawal or if she had not been using any type of contraception at all [26–28].

#### Independent variables

Seventeen independent variables that were theoretically and empirically related to contraceptive usage were considered in this study [27–33]. The variables comprised age, occupation, place of residence, region of residence, marital status, education, partner’s education, wealth, age at first sex, age at first childbirth, total children ever born, number of living children, husband’s desire for children, decision maker on contraception, frequency of listening to radio, frequency of watching television, and frequency of reading newspaper or magazine. Some of these variables were recoded for meaningful and easy interpretation of results. Age at first sex and age at first childbirth were recoded as 1 = < 18, 2 = 18–24, and 3 = 25+. Total children ever born was coded as 1 = 1–2, 2 = 3–4, and 3 = 5 and above. Number of living children was coded as 1 = No Child, 2 = 1–2, 3 = 3–4, and 4 = 5 and above.

### Statistical analysis

Both descriptive and inferential analyses were conducted. The descriptive analysis involved the use of frequencies and percentages to describe the study sample and the prevalence of modern contraceptive usage across all the independent variables. After that, two logistic regression models were built and the results were reported as crude and adjusted odds ratios (see Table 2). The model fitness specification was done with the Hosmer-Lemeshow test while multicollinearity was checked using the variance inflation factor (VIF) which showed no evidence of multi-collinearity. We applied sample weight to take care of areas that were under-sampled and also those that were over-sampled. The SVY command was also used to take care of the multi-stage sampling nature of the survey. STATA Version 14.2 for MacOS was used to carry out the analyses, and statistical significance was declared at *p*-value less than 0.05.

## Results

### Prevalence of modern contraceptive use among women in union in Papua New Guinea

**Fig. 1:**
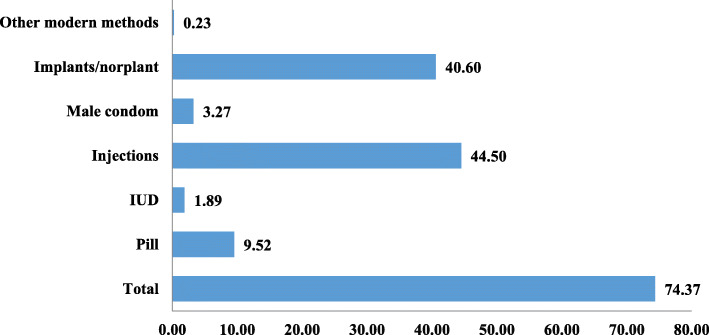
Prevalence of modern contraceptive use among women in union in Papua New Guinea

Findings on the prevalence of modern contraceptive use are presented in Fig. 1. Injectables were the most widely used modern contraceptive, ensued by implants/Norplant, marked by 44.50 and 40.60% respectively. The least reported was other modern methods (0.23%). In all, 74.37% of the women were using modern contraceptives.

### Socio-demographic characteristics of women in sexual union in Papua Guinea and modern contraceptives usage

Modern contraceptive use with respect to socio-demographic characteristics have been presented in Table 1. Contraceptive use dominated among women aged 20–24 (83.0%). Nearly 83.3% of women in skilled manual labour were using modern contraceptives. Modern contraceptive use was high among urban residents (76.9%). Majority (86.2%) of those in the Highlands region were using modern contraceptives. Modern contraceptives was high among married women (74.4%). Women with primary education dominated in modern contraceptive use (75.3%). Modern contraceptive use was high among women whose partners had no formal education (81.5%). Use of modern contraceptives was high for poorer women (78.2%). Modern contraceptive use stood at 76.4% for those below 18 years. Modern contraceptive use was well pronounced (78.3%) among those under age 18. Modern contraceptive use was high for women with 3–4 births (76.5%). Modern contraceptive use was high among women with 1–2 children living (76.5%). A greater section of the women (47.1%) reported that they and their husbands desired children. Meanwhile, modern contraceptive use was common for women whose husbands alone desired children. We found that modern contraceptive use was commonly reported among those who read at least once a week (75.3%) as well as those who listened to radio at least once a week. Similarly, 78.1% of those who watched television at least once a week made use of modern contraceptives. Most of the women made joint decision on contraceptive with their husbands (58.3%). Nonetheless, 77.9% of those who made solo decisions on contraceptives reported usage.

**Table 1:** Socio-demographic characteristics of women in union in Papua New Guinea and modern contraceptives usage (*N* = 2345)

Variable	Weighted N	Weighted %	Modern Contraceptive use	
			No n(%)	Yes n(%)
**Age**				
15–19	50	2.1	8 (16)	42 (84.0)
20–24	387	16.5	66 (17.1)	321 (83.0)
25–29	581	24.8	107 (18.4)	474 (81.6)
30–34	549	23.4	146 (26.6)	403 (73.4)
35–39	404	17.2	119 (29.5)	285 (70.5)
40–44	249	10.6	93 (37.4)	156 (62.7)
45–49	125	5.3	62 (49.6)	63 (50.4)
**Respondent’s occupation**				
Not working	1398	59.6	375 (26.8)	1023 (73.2)
Professional /technical/managerial	158	6.7	64 (40.5)	94 (59.5)
Clerical	57	2.4	13 (22.8)	44 (77.2)
Sales	128	5.5	30 (23.4)	98 (76.6)
Agricultural- self employed	317	13.5	58 (18.3)	259 (81.7)
Agricultural – employee	42	1.8	12 (28.6)	30 (71.4)
Services	227	9.7	46 (20.3)	181 (79.7)
Skilled manual	18	0.8	3 (16.7)	15 (83.3)
**Residence**				
Urban	640	27.3	148 (23.1)	492 (76.9)
Rural	1705	72.7	453 (26.6)	1252 (73.4)
**Region**				
Southern region	751	32.0	149 (19.8)	602 (80.2)
Highlands region	462	19.7	64 (13.9)	398 (86.2)
Momase region	548	23.4	120 (21.9)	428 (78.1)
Islands region	584	24.9	268 (45.9)	316 (54.1)
**Current marital status**				
Married	2015	85.9	515 (25.6)	1500 (74.4)
Living with partner	330	14.1	86 (26.1)	244 (73.9)
**Highest educational level**				
No education	389	16.6	99 (25.5)	290 (74.6)
Primary	1141	48.7	282 (24.7)	859 (75.3)
Secondary	698	29.8	175 (25.1)	523 (74.9)
Higher	117	5.0	45 (38.5)	72 (61.5)
**Husband/partner’s education level**				
No education	260	11.1	48 (18.5)	212 (81.5)
Primary	1037	44.2	292 (28.2)	745 (71.8)
Secondary	820	35.0	186 (22.7)	634 (77.3)
Higher	228	9.7	75 (32.9)	153 (67.1)
**Wealth index**				
Poorest	268	11.4	74 (27.6)	194 (72.4)
Poorer	335	14.3	73 (21.8)	262 (78.2)
Middle	436	18.6	124 (28.4)	312 (71.6)
Richer	637	27.2	158 (24.8)	479 (75.2)
Richest	669	28.5	172 (25.7)	497 (74.3)
**Age at first sex**				
< 18 yrs	815	34.8	192 (23.6)	623 (76.4)
18–24	1364	58.2	347 (25.4)	1017 (74.6)
25+	166	7.1	62 (37.4)	104 (62.6)
**Age at first childbirth**				
< 18	419	17.9	91 (21.7)	328 (78.3)
18–24	1539	65.6	376 (24.4)	1163 (75.6)
25+	387	16.5	134 (34.6)	253 (65.4)
**Total children ever born**				
1–2	925	39.5	218 (23.6)	707 (76.4)
3–4	851	36.3	200 (23.5)	651 (76.5)
5+	569	24.3	183 (32.2)	386 (67.8)
**Number of living children**				
No child	13	0.6	6 (46.2)	7 (53.8)
1–2	962	41.0	226 (23.5)	736 (76.5)
3–4	857	36.6	206 (24)	651 (76)
5+	513	21.9	163 (31.8)	350 (68.2)
**Husband’s desire for children**				
Both want same	1104	47.1	316 (28.6)	788 (71.4)
Husband wants more	424	18.1	96 (22.6)	328 (77.4)
Husband wants fewer	80	3.4	16 (20)	64 (80)
Don’t know	737	31.4	173 (23.5)	564 (76.5)
**Frequency of reading newspaper or magazine**				
Not at all	1340	57.1	352 (26.3)	988 (73.7)
Less than once a week	551	23.5	137 (24.9)	414 (75.1)
At least once a week	454	19.4	112 (24.7)	342 (75.3)
**Frequency of listening to radio**				
Not at all	1421	60.6	398 (28)	1023 (72)
Less than once a week	471	20.1	105 (22.3)	366 (77.7)
At least once a week	453	19.3	98 (21.6)	355 (78.4)
**Frequency of watching television**				
Not at all	1721	73.4	457 (26.6)	1264 (73.4)
Less than once a week	255	10.9	63 (24.7)	192 (75.3)
At least once a week	369	15.7	81 (22)	288 (78)
**Decision maker on contraception**				
Mainly respondent	659	28.1	146 (22.2)	513 (77.8)
Mainly husband, partner	294	12.5	83 (28.2)	211 (71.8)
Joint decision	1367	58.3	365 (26.7)	1002 (73.3)
Other	25	1.2	7 (28)	18 (72)

Source: 2016–18 PNG DHS

### Predictors of modern contraceptive utilization among women in unions in Papua New Guinea

We present the predictors of modern contraceptive use in Table 2. Relative to women aged 45–49, those aged 15–19 had the highest odds of modern contraceptive use [AOR = 7.425, 95% CI = 2.853, 19.32]. In both models, residents of the Highland region had higher odds of modern contraceptive use, compared to those in the Southern region [AOR = 1.521, 95% CI =1.086, 2.131] but the reverse was the situation for residents of the Islands region [AOR = 0.291, 95% CI = 0.224, 0.377]. Women whose husbands had higher education had less odds of modern contraceptive use, relative to those whose husbands had no formal education [AOR = 0.531, 95% CI = 0.318, 0.886]. Whereas professional/technical/managerial workers had lower odds of modern contraceptive use [AOR = 0.643, 95% CI = 0.420, 0.986], higher odds were noted for women who were self-employed in the agricultural sector, relative to women who were not working [AOR = 1.710, 95% CI = 1.218, 2.400]. Women with no living child were less likely to use modern contraceptives compared to those with five or more children [AOR = 0.213, 95% CI = 0.0498,0.911]. Conversely, women who did not know their partners’ desire for children were more likely to use modern contraceptives AOR = 1.286 95% CI = 1.015,1.630].

**Table 2:** Predictors of modern contraceptive utilization among women in union in Papua New Guinea (*N* = 2345)

Variable	COR[95%CI]	AOR [95%CI]
**Age**		
15–19	5.167***[2.245,11.89]	7.425***[2.853,19.32]
20–24	4.786***[3.084,7.428]	6.312***[3.591,11.10]
25–29	4.360***[2.897,6.560]	5.333***[3.281,8.669]
30–34	2.716***[1.824,4.046]	2.803***[1.811,4.338]
35–39	2.357***[1.563,3.554]	2.352***[1.544,3.584]
40–44	1.651*[1.069,2.549]	1.500 [0.966,2.330]
45–49	**Ref**	**Ref**
**Marital status**		
Married	1.027 [0.787,1.339]	–
Cohabiting	**Ref**	**Ref**
**Residence**		
Urban	1.203 [0.972,1.488]	–
Rural	**Ref**	**Ref**
**Region**		
Southern region	**Ref**	**Ref**
Highlands region	1.539** [1.119,2.118]	1.521* [1.086,2.131]
Momase region	0.883 [0.674,1.157]	1.03 [0.773,1.373]
Islands region	0.292***[0.229,0.372]	0.291***[0.224,0.377]
**Highest educational level**		
No education	**Ref**	**Ref**
Primary	1.04 [0.798,1.355]	1.32 [0.985,1.769]
Secondary	1.02 [0.767,1.357]	1.3 [0.912,1.853]
Higher	0.546** [0.353,0.845]	1.086 [0.577,2.047]
**Husband/partner’s educational level**		
No education	**Ref**	**Ref**
Primary	0.578**[0.411,0.813]	0.737 [0.505,1.076]
Secondary	0.772 [0.542,1.099]	0.881 [0.585,1.325]
Higher	0.462***[0.304,0.701]	0.531* [0.318,0.886]
**Wealth status**		
Poorest	**Ref**	**Ref**
Poor	1.369 [0.943,1.988]	–
Middle	0.96 [0.684,1.347]	–
Rich	1.156 [0.838,1.597]	–
Richest	1.102 [0.801,1.516]	–
**Employment**		
Not working	**Ref**	**Ref**
Professional/technical/managerial	0.538***[0.384,0.756]	0.643* [0.420,0.986]
Clerical	1.241 [0.661,2.329]	1.082 [0.519,2.257]
Sales	1.197 [0.782,1.833]	1.299 [0.822,2.054]
Agricultural - self employed	1.637** [1.203,2.228]	1.710**[1.218,2.400]
Agricultural - employee	0.916 [0.464,1.809]	1.246 [0.551,2.817]
Services	1.442*[1.022,2.036]	1.385 [0.944,2.031]
Skilled manual	0.855 [0.220,3.325]	0.646 [0.188,2.223]
**Total children ever born**		
1–2	1.538***[1.219,1.940]	1.333 [0.496,3.585]
3–4	1.543***[1.218,1.955]	1.485 [0.788,2.800]
5+	**Ref**	**Ref**
**Number of living children**		
No child	0.543 [0.180,1.642]	0.213*[0.0498,0.911]
1–2	1.517***[1.195,1.925]	0.469 [0.175,1.257]
3–4	1.472** [1.154,1.877]	0.665 [0.352,1.258]
5+	**Ref**	**Ref**
**Age at first sex**		
< 18 yrs	1.934***[1.358,2.755]	0.961 [0.616,1.501]
18–24	1.747**[1.247,2.448]	1.158 [0.777,1.727]
25+	**Ref**	**Ref**
**Age at first childbirth**		
< 18	1.909***[1.396,2.610]	0.892 [0.568,1.401]
18–24	1.638***[1.289,2.082]	0.992 [0.722,1.363]
25+	**Ref**	**Ref**
**Partner desire for children**		
Both want same	**Ref**	**Ref**
Husband wants more	1.370*[1.054,1.781]	1.318 [0.984,1.765]
Husband wants fewer	1.604 [0.913,2.817]	1.433 [0.764,2.689]
Don’t know	1.307*[1.055,1.620]	1.286* [1.015,1.630]
**Decision maker for using contraception**		
Mainly respondent	1.280*[1.028,1.594]	1.2 [0.934,1.542]
Mainly husband, partner	0.926 [0.699,1.226]	0.923 [0.683,1.247]
Joint decision	**Ref**	**Ref**
Other	0.937 [0.388,2.261]	0.876 [0.333,2.303]
**Frequency of listening to radio**		
Not at all	**Ref**	**Ref**
Less than once a week	1.356* [1.060,1.734]	1.320*[1.001,1.741]
At least once a week	1.409** [1.096,1.813]	1.409* [1.048,1.895]
**Frequency of reading newspaper or magazine**		
Not at all	**Ref**	**Ref**
Less than once a week	1.077 [0.857,1.353]	–
At least once a week	1.088 [0.851,1.391]	–
**Frequency of watching television**		
Not at all	**Ref**	**Ref**
Less than once a week	1.102 [0.813,1.493]	–
At least once a week	1.286 [0.983,1.682]	–
**N**		2345

Source: 2016–18 PNG DHS

Exponentiated coefficients; 95% confidence intervals in square brackets

^*^ *p* < 0.05

^**^ *p* < 0.01

^***^ *p* < 0.001

*ref.* reference, *CI* confidence interval, *COR* Crude Odds Ratio, *AOR* Adjusted Odds Ratios

## Discussion

This study investigated the prevalence and drivers of modern contraceptive use in Papua New Guinea, a country with a history of low modern contraceptive use amidst high maternal mortality [34]. In all, we found that at least seven out of ten of the women were using modern contraceptives (74.37%). Injectables were the commonly used modern contraceptives. To a larger extent, modern contraceptive use in Papua New Guinea has levitated at a faster pace. When Marie Stopes [34] started its operations in the country in 2006, contraceptive use was a little above 25% and this seems to have tripled in less than two decades.

The modern contraceptive prevalence observed exceeds modern contraceptive prevalence in the Oceanian region (58%) [35]. The prevalence is analogous to the contraceptive prevalence in Northern America (74%) [35]. Just as observed, the 2006 Demographic and Health Survey of Papua New Guinea noted that injectables were the leading modern contraceptives (36.9%) relative to any other method [36]. A Nigeria-based study that employed time-trend analysis on data ranging between 2000 and 2014 similarly noted that injectables were the leading modern contraceptives (40.7%) [37]. The underlying factors for consistency in preference for injectables in Papua New Guinea are, however, not revealed and a qualitative study will be useful in this regard.

Women aged 15–19 had the highest odds of modern contraceptive use, relative to 45–49 aged women. This finding might rationalize the essence of the ongoing advocacies and initiatives by the Marie Stopes International and its sister organisations. Reporting from a Ugandan population-based study, Rutaremwa et al. [38] also realised a descending trend in modern contraceptive use in relation to age. Since 2006, the Marie Stopes International has focused on making reproductive health services easily accessible to young persons, since this category of people was under-served in the past. In 2018, more than one third of the persons that were served by the organisation (37%) were below 25 years. Practically, institution of a specialised hotline, initiation of youth-friendly services, and collaboration with a revered leading religious institution contributed significantly in achieving this milestone [34].

Residents of the Highland region had higher odds of modern contraceptive use, compared to those in the southern region, but the reverse was the situation for residents of the Islands region. Over the years, several reproductive health policies have been instituted since 1908 [39], the most recent of which are the *National Family Planning Policy (2014)*, *National Youth and Adolescent Health Policy (2014), National Sexual Reproductive Health Policy (2014)*, and the *National Health Sector Gender Policy (2014*) [39]. If in spite of all these, women in some parts of the country exhibit high tendency of modern contraceptive use than others, then the supply or distribution of modern contraceptives should be reconsidered so as to ensure equal access to all women irrespective of geographical location.

Women whose husbands had higher education had less odds of modern contraceptive use, relative to those whose husbands had no formal education. These findings are inconsistent with previous evidence [37, 38, 40]. Education is noted to be a pathway to empowerment over a woman’s reproductive health and her holistic wellbeing, and increases awareness of benefits and demerits of contraceptives [38, 41]. These notwithstanding, our finding suggests that highly educated women may be pursuing career and professional objectives and, as such, have limited or no time for contraceptive, relative to low or non-educated women who may not be engaged in highly competitive professions that warrant more commitment.

Whereas professional/technical/managerial workers had lower odds of modern contraceptive use, higher odds were noted for women who were self-employed in the agricultural sector, relative to women who were not working. These variations may be due to preferences of these women. Whilst modern contraceptive is the most preferred [42], some women may prefer either traditional or natural methods for reasons best known to them [43, 44]. We noticed higher odds among women with 3–4 births. However, this observation lost its significance when fitted in the multivariate model variable, which implies that some critical traits of a woman can offset the implication of parity/birth order on her prospects of using modern contraceptives.

Listening to radio at least once a week was associated with higher odds of contraceptive use, compared to not listening at all. Media exposure, such as listening to radio, has similarly been reported to boost modern contraceptive use in Uganda [38]. Some researchers have also contended that mass media enhances demand of services and induces long-term change in behaviour via information, education, and communication initiatives [45].

### Strengths and limitations

The study has some limitation worth mentioning. The use of modern contraceptives is subject to social, cultural, and religious interpretations. As a result, a woman may be using contraceptives clandestinely from her partner or family member and may, therefore, not have the courage to report that she uses modern contraceptive [32]. This situation will eventually lead to under-reporting of modern contraceptive use. Secondly, since the information on modern contraceptives and the independent variables was gathered concurrently, attribution cannot be made [32]. These notwithstanding, the study has some compelling strengths. It provides current information on the type and prevalence of modern contraceptive use in Papua New Guinea. Additionally, the rigour of the methodological approach and representativeness of the survey render its findings and recommendations generalizable to the country and replicable to other Oceania countries.

## Conclusion

We strived to investigate the prevalence of modern contraceptive use and underlying factors in Papua New Guinea, a country in the Oceania region. Of the 2345 participants, we found that the majority of the women were using modern contraceptives, with the commonest method being injectables. Age, region of residence, partners education, employment, partner desire for children, frequency of listening to radio are associated with modern contraceptive usage. Tailored reproductive health care should be developed for women who are disadvantaged when it comes to the use of modern contraceptives in order to boost modern contraceptive use among them. Further investigation is needed to unravel the motivation for injectables among these women.

## Data Availability

The dataset can be accessed at https:// https://dhsprogram.com/data/dataset/Papua-New-Guinea_Standard-DHS_2017.cfm?flag=0

## Abbreviations

COR: Crude Odds Ratio
AOR: Adjusted Odds Ratio
CI: Confidence Interval
PDHS: Papua New Guinea Demographic and Health Survey
NSO: National Statistical Office
